# Densification and Strengthening of Aerogels by Sintering Heat Treatments or Plastic Compression

**DOI:** 10.3390/gels4010012

**Published:** 2018-01-31

**Authors:** Thierry Woignier, Laurent Duffours

**Affiliations:** 1 Aix Marseille University, University Avignon, Centre National de la Recherche Scientifique (CNRS), Institut de Recherche pour le Developpement (IRD), Institut Méditerranéen de Biodiversité et d’Ecologie (IMBE), Marseille, 13397, France; 2 Institut Méditerranéen de Biodiversité et d’Ecologie, Campus Agro Environmental Caraïbes, B.P. 214 Petit Morne, 97232 Le Lamentin, France; 3 Prime Verre, 34090 Montpellier, France; laurent.duffours@primeverre.com

**Keywords:** aerogels, sintering, isostatic pressure, plastic hardening, elastic properties

## Abstract

Due to their broad range of porosity, aerogels are suited to various applications. The advantages of a broad range of porosity are used directly, for example, in thermal and acoustic insulation, as materials for space applications or in catalysers. However, an overly high pore volume can also be a drawback, for example, in a glass precursor and host matrix. Fortunately, aerogel porosity can be tailored using sintering or isostatic compression. Sets of silica aerogels—sintered and compressed aerogels—have been studied with the objective of comparing these different densification mechanisms. We focus on the mechanical changes during the two processes of densification.

## 1. Introduction

Aerogels are fascinating materials because of their exceptional properties, such as low thermal conductivity, low densities and refractive index, low sound velocity, etc. [1,2,3]. These properties are related to the large porosity which can be higher than 99% [1,2]. This large pore volume can also be used as a host matrix to obtain different kinds of materials such as multicomponent aerogels, binary glasses, composites, and materials with gradient properties [1,4,5]. For this purpose, some physical properties that depend on the aerogel porosity (permeability, diffusion, mechanical properties) are important because they affect the transport of the chemical species, the pore volume and the brittleness of the matrix. So, the control of the porosity features and microstructure of the solid network, such as pore volume, density, and pore size distribution, is important.

There are different ways in which to synthesise sets of samples with a tailored porosity (or bulk density). Generally, the pore volume is adjusted by the organosilicon content (tetramethoxysilane (TMOS) in the solution before gelation [1,2,6]. However, the pH of the solution could also change the aggregation mechanisms of the monomers and lead to different microstructure and porosity [7]. The temperature and duration of the aging before the supercritical step also affects the pore volume. However, these different synthesis parameters—organosilicon content, pH, aging and temperature—will control the bulk density in a restricted range and the final bulk density is generally not higher than 0.4 g·cm^−3^. For certain applications, the porosity of the aerogels should be lower than the usual porosity of aerogels (80–95%). For example, when aerogels are used as a host matrix in an impregnation process, we have shown that the aerogel should have a pore volume close to 50% (bulk density close to 1 g·cm^−3^), before the impregnation step. To attain this density, the sintering process allows the porous structure to be progressively collapsed, thus increasing the mechanical properties which enables resistance to the capillary forces [5].

Another possible way to control the porosity features is by isostatic pressure. Silica aerogels are generally described as purely elastic materials and no plastic deformation has been reported when an aerogel is subjected to a tension load [8,9]. However, it has been shown [10,11,12] that if the aerogel is subjected to strong isostatic pressure, an irreversible strain is observed, characteristic of plastic behaviour; volume shrinkages higher than 50% can also be observed.

In this paper, we report sintering and compression experiments performed on silica aerogels. Different kinds of information will be analysed: the evolution of the dimensional shrinkage and bulk density with the densification parameters (temperature, pressure, duration); the evolution of the elastic and mechanical properties due to sintering or isostatic pressure. The elastic and mechanical properties will give insight into the evolution of the connectivity.

## 2. Results and Discussion

### Aerogel Sintering by Heat Treatments

Thermal treatments allow the aerogel to be converted into porous glasses and/or fully dense silica glass and the final density of the sintered aerogels will depend on the thermal treatments applied. Sintering is a process by which the surface area of a material is decreased by mass transport [13]. For amorphous materials, viscous flow is important, because it is much faster than the densification process resulting from diffusion.

**Figure 1:**
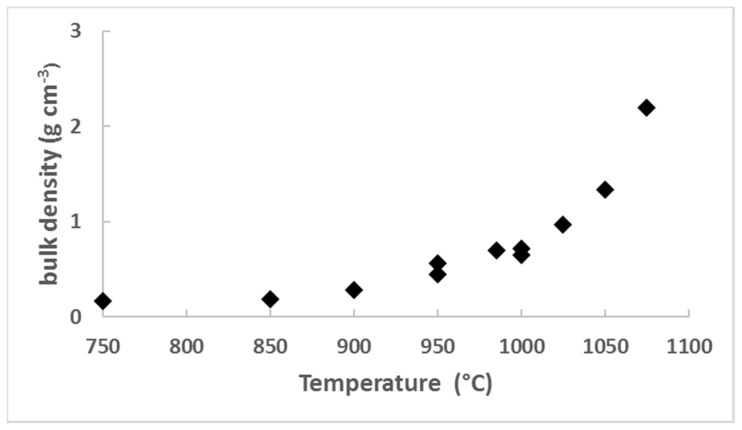
Bulk density versus the temperature of the heat treatment (2 h). The initial bulk density is 0.16 g·cm^−3^.

**Figure 2:**
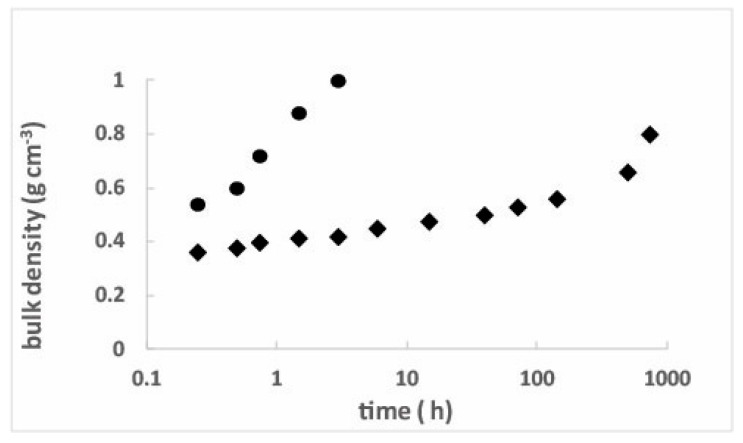
Bulk density versus time: isothermal heat treatment at 800 °C (♦) and 950 °C (●). The initial bulk density is 0.32 g·cm^−3^.

Figure 1 shows the sintering measurements taken at several temperatures in the 750–1050 °C range (2 h). The data show the large increase in the density (porosity decrease) with the temperature. In the temperature range 1050–1075 °C, the pore volume is completely collapsed and the bulk density is close to 2.2 g·cm^−3^: the silica glass density. Experiments performed at 800 and 950 °C, as a function of time, allow a more precise control of the density by sintering (Figure 2). It is thus possible to synthesise sintered aerogels (porous glasses) in the density range 0.18–1 g·cm^−3^.

Scherer [13] proposed a model describing the viscous flow sintering of amorphous material over a wide range of porosity (0–95%). The set of aerogels studied confirms previous data [5]: the sintering occurs by viscous flow and the isothermal densification kinetics of aerogels fit Scherer’ model.

#### 2.1.1. Effect of Sintering on Mechanical and Elastic Properties

**Figure 3:**
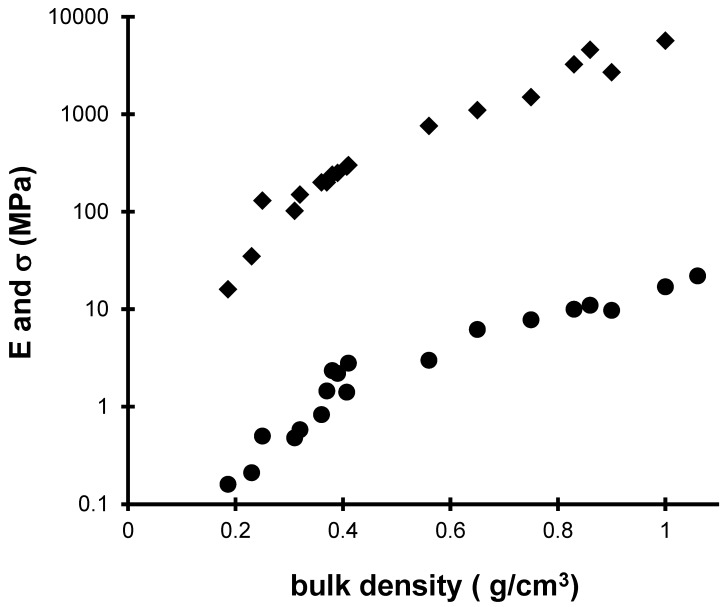
*E* (♦) and *σ* (●) evolution for sintered aerogels versus the bulk density for sintered aerogels.

During the sintering heat treatments, the microstructure of the aerogel is changed and the elastic and mechanical properties are enhanced. Figure 3 presents the evolution of the Young (*E*) and the rupture moduli (*σ*) as a function of the density produced by sintering.

The main feature of these curves is the very large increase (10^2^–10^3^) in the elastic and mechanical properties over the density range. The strengthening of the material is directly related to the decrease in the pore volume [14,15] but also to structural changes [16,17]. In the density range 0.18–0.4 g·cm^−3^, the E and σ values of sintered aerogel are higher than for not sintered aerogels with the same bulk density [18]. We can conclude that the heat treatment has induced an increase in the connectivity or the size of the necks between particles. Organic species and silanol groups are replaced by new siloxane bonds, increasing the connectivity and thus the mechanical features. The sintering reduces the whole sample volume, eliminating the macro and microporosity [19]. Besides the elimination of pores, the heat treatment increases the network connectivity [18].

#### 2.1.2. Densification of Silica Aerogels by Isostatic Pressure

Different works [10,11,12] have shown that if an aerogel is subjected to a compressive load, the solid network initially behaves elastically, until the strain is no longer proportional to the stress (yield strength). After the stress release, an irreversible strain is observed, characteristic of a plastic behavior. Owing to the plastic shrinkage, the material shrinks progressively due to pore collapse.

**Figure 4:**
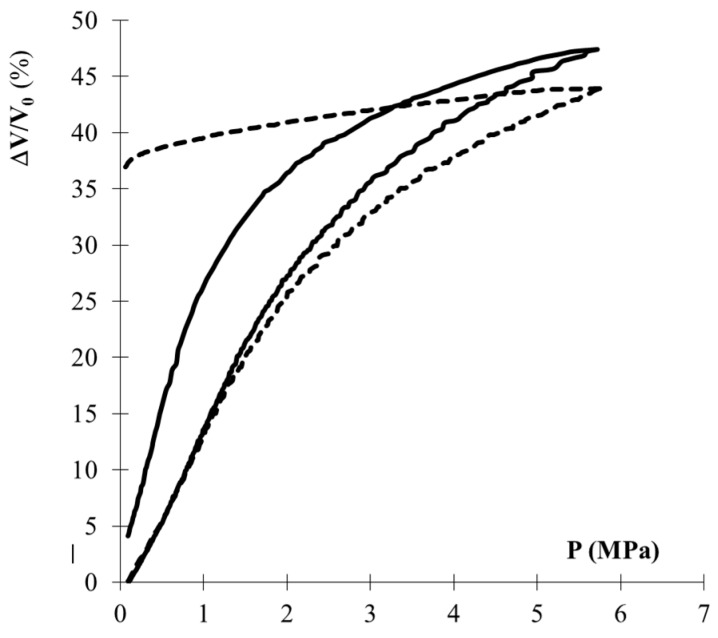
Typical volume strain (Δ*V/V*_0_) versus applied pressure. (Continuous line is as-received AER60; dotted line is oxidised AER60).

The typical curve of the relative volume shrinkage, (∆*V/V*_0_), is shown in Figure 4 as a function of applied pressure. If the applied pressure is lower than the yield strength (*σ*_el_), the sample deforms elastically; the volume strain is proportional to the stress applied and, after the pressure release, the sample recovers its initial volume. When the pressure applied is higher than the yield strength (≈1–1.5 MPa in this case), the behavior is no longer elastic and part of the volume strain is irreversible. The irreversible volume shrinkage (∆*V/V*_0_)_pl_ characterises the magnitude of the plastic strain and *σ*_el_ delimits the elastic range. Thus, as a function of the pressure applied during the run, ρ increases and the porosity decreases. Correlatively, its mechanical features *K*, *E* and *σ*_el_ are modified. It is then possible to determine new *K*, *E* and *σ*_el_ values for the compressed material, by a new run. Thus, from such experiments, one can characterise the magnitude of the irreversible shrinkage and the associated evolution of the elastic and mechanical properties (*K*, *E* and *σ*_el_).

Macroscopically, pressure and temperature apparently result in the same effect: they induce pore collapse and a density increase. One is tempted to draw a parallel between compression and the high temperature sintering mechanism. In the following, the shrinkage by compression is compared to the shrinkage by sintering.

In Table 1 and Table 2, the volume shrinkage is plotted as a function of applied pressure for two different samples: AER30 and AER50 respectively. Although the differences in TMOS content seem to be slight, the samples’ plastic behaviour is strongly affected. AER30 is easily compressed at low pressure but the volume shrinkage reaches a plateau at a pressure of about 12 MPa. AER50 shrinks continuously up to a plateau at 60–80 MPa. The shrinkage limit can indeed be attributed to the rigidity increase of the network. From knowledge of the relative volume shrinkage, one can calculate the density evolution. The density increase is small for the lightest sample (AER30) and larger for the denser one (AER50). This counterintuitive result can be explained by the elastic moduli *K* and *E* and the yield strength *σ*_e**l**_ of evolutions as a function of density (Table 1 and Table 2).

These two tables evidence that stiffening and strengthening are very fast for the lightest sample (AER30) and much slower for sample AER50. This explains why further volume shrinkage is more difficult for the AER30 sample. We must also underline that, during the first steps of densification of the sample AER50, *K*, *E* and *σ*_el_ exhibit a slow decrease.

To confirm these results, we measured the loss of connectivity by more accurate measurements. The sound velocity is investigated by ultrasound experiments [20]. Sound velocity *V* is very sensitive to the connectivity of the solid network and a small variation in the network connectivity will strongly affect the sound velocity. Table 3 and Table 4 show that the sound velocity and the elastic moduli (*K* and *E*) of the sintered and compressed sets exhibit two different behaviors.

For the sintered set, the sintering is accompanied by a monotonic stiffening of the material; the sound velocity of the sample of 0.33 g·cm^−3^ is three times lower than the one of 0.75 g·cm^−3^. On the other hand, the compressed aerogels show a lowering of *V*, between 0.33 and 0.42 g·cm^−3^, characterizing a loss in the network connectivity. In the second part of the table (0.42–0.55 g·cm^−3^), the compressed set shows an increase in *E*, leading to the conclusion that after the connectivity loss, compression likely induces the formation of siloxane bonds.

The silica aerogel microstructure is usually described as a hierarchical network in the length scale 1 to 100 nm. The structure is the result of an aggregation mechanism; the silica beads (≈1 nm) build the aggregates [7,21]. For sintered samples, the densification tends to contract the aggregates and reduces the sample volume. The densification proceeds by coalescence of small particles into larger ones. The local sintering has two effects: it shrinks the aggregates and increases the connectivity. On the other hand, in the case of densification by compression, the aggregates interpenetrate each other; their periphery is changed, but their internal structure is not affected. This rearrangement is reasonable, taking into account the process which stresses the samples by isostatic external pressure. During compression, because the solid is not viscous, such a restructuring should induce strain and local disconnection in the network.

To allow the motion of the aggregates, part of the links is broken and the whole connectivity of the network is lowered (*V* decreases). However, because the aggregates touch and interpenetrate, silanol groups (SiOH) can polycondense and the formation of new siloxane bonds will likely increase the network connectivity (*V* further increases).

The silanol content is thus an important parameter. Figure 4 shows the plastic shrinkage versus the pressure applied, for two samples with the same bulk density, but with different silanol content: the oxidised aerogel has a silanol content larger than the as-prepared sample [5]. The plastic shrinkage of the oxidised sample is twice that of the as-prepared aerogel: as expected, the OH content favours the shrinkage. The silanol groups condense, freezing the strained structure. For as-prepared aerogels, only a few siloxane bonds are created and after the pressure release the sample partly recovers its initial volume.

The fast stiffening and strengthening observed in the case of the lightest samples can also be explained by the effect of the silanol content. AER30 exhibits a larger specific surface area and consequently a higher OH content. The larger pores and higher OH content of AER30, compared to AER50, favour the motion of aggregates and their ability to form new links.

## 3. Conclusions

Aerogels are probably the solid materials with the highest pore volume ever synthesised. The possibility to sinter aerogels into dense silica glass allows porous solid materials to be synthesised over a large range of porosity (95 to 0%). Another way to decrease the porosity is the isostatic pressure. The important parameters of this densification by compression are the elastic properties of the material (which allow, or not, the sample strain), the macroporous volume (which allow the aggregates to move) and also the silanol content (which favours the freezing of the strained structure by the formation of siloxane bonds Si–O–Si).

This method represents a new way to synthesise porous glasses at room temperature and to prepare composite material even in the case that one of the components is not able to tolerate high temperatures (dye laser, biological species, drugs, etc.). This densification process, by eliminating the larger pores without changing the internal aggregates’ structure, allows different kinds of porous glasses to be synthesised with different physical properties, such as acoustical properties, thermal conductivity or permeability.

## 4. Materials and Methods

### 4.1. Materials Synthesis

The silica gels selected in this study were made from tetramethoxysilane (TMOS), hydrolysed under neutral conditions (4 moles of distilled water per mole of TMOS). The TMOS–ethanol–H_2_O mixture was stirred (30 min) and aged one week at room temperature. The solid volume fraction was adjusted from the TMOS content diluted in the initial solution. The gels are transformed into aerogels by supercritical drying performed at 305 °C and 13 MPa with a heating rate close to 0.25 °C/min. When the pressure of 13 MPa was reached and after a period of 30 min to permit equilibration of temperatures and pressure, the autoclave was depressurised isothermally to atmospheric pressure by condensation of the superfluid outside of the autoclave; the depressurization rate was close to 0.15 MPa/min. The aerogels were labeled as AERy, where y represents the TMOS content. Just after drying, aerogels exhibited hydrophobic properties. A thermal treatment at 350 °C for 12 h allowed their surface organic radicals to be removed. Bulk density (*ρ*) was determined by measuring the samples’ weight and dimensions; porosity was calculated from the bulk density and the skeletal density close to 2 g·cm^−3^. The bulk density varied between 0.16 and 0.32 g·cm^−3^. After oxidation, ρ did not vary significantly (less than 3%), because the organic radicals were replaced by hydroxyls and adsorbed water.

### 4.2. Densification Procedure

The sintering of silica aerogels, proceeded by viscous flow at high temperature, has been described previously [5,19]. Depending on the duration of the heat treatment, the pores collapse and the bulk density increases to the density of the silica glass, i.e., 2.2 g·cm^−3^. In this study, the sintering is performed in air, in the temperature range 750–1050 °C. The sintered aerogel samples covered the density range between 0.18 and 1.1 g·cm^−3^ (porosity within the range 98–50%).

Isostatic compression experiments were performed using Hg porosimetry (Carlo Erba Porosimeter 2000—Rodano, Milano, Italy) on out gassed (10^−2^ torr, 1 h) monolithic samples (<3 cm^3^). Hg pressure can be varied from 0.1 to 100 MPa. In such experiments, as mercury cannot penetrate the pores, the aerogel is isostatically compressed [10,11,12]. In a previous study [11], the aerogels were placed into an impermeable membrane or directly in the Hg. The authors showed that measurement of companion samples (with and without the membrane) yielded the same results.

The samples were compressed to a given pressure at a rate of 1 MPa/min and after depressurization, the irreversible volume shrinkage was precisely measured from the mercury level using a special optical device (cathetometer, SIMPO-Bouty, Paris, France). The irreversible volume shrinkage (∆*V/V*_0_)_pl_ characterises the magnitude of the plastic strain. The compressed aerogel samples of this study covered a density between 0.18 and 0.8 g·cm^−3^ (porosity within the range 98–55%).

### 4.3. Measurements of Elastic and Mechanical Properties

The Young elastic modulus (*E*) and the rupture modulus (*σ*) of the samples were measured by a 3-point bending technique using an Instron 1196 mechanical testing machine with crosshead speed which could be varied from 10 µm/min to 100 mm/min [8,16]. The dimensions of the gel samples were 40 × 5 × 3 mm^3^. The samples were placed on a span of 30 mm (the span to thickness ratio was 10).

During pressure increase by isostatic compression experiments, the sample exhibited a volume strain directly related to its compressibility. The elastic bulk modulus, K(*P*) can be calculated using: K(*P*) = −V(*P*) d*P*/d*V* and *σ*_el_ is estimated from the limit of the linear part [12]. From the K(*P*) and the Poisson’s ratio (*ν*), we calculated E(*P*) using the relation E(*P*) = 3(1 − 2*ν*) K(*P*); for aerogels, the Poisson’s ratio was close to 0.18 [7].

One method to determine the sound velocity in a material consists of determining the propagation delay between faces of a sample in which an impulse wave propagates. In this study, the setup consisted of two identical transducers (emitter and receiver). The distance between the emitter and receiver was around 30 mm with an accuracy of 0.1 mm. Measurements were taken with a set of transducers (Panasonic V101, 500 kHz, Kadoma-shi, Japan) [20]. From the sound velocity of longitudinal waves (*V*) and the Poisson’s ratio (*ν*), we calculated E using the relation: E = *ρV*^2^(1 − 2*ν*)(1 + *ν*)/(1 − *ν*).

## Figures and Tables

**Table 1:** Irreversible volume shrinkage (Δ*V/V*_0_)_pl_, bulk density *ρ*, elastic moduli *K* and *E* and yield strength *σ*_el_ versus applied pressure *P* for AER30.

*P* (Mpa)	(Δ*V/V*_0_)_pl_ (%)	*ρ* (g·cm^−3^)	*K* (Mpa)	*E* (Mpa)	*σ*_el_ (Mpa)
0.1	0	0.16	20	38.4	0.3
1.22	1.8	0.18	27	51.8	0.3
2.38	17.8	0.19	44.2	84.8	0.52
3.6	22.9	0.2	68.1	130.7	0.57
5.5	27.8	0.21	92.6	177.7	0.73
5.98	30.6	0.23	118.9	228.2	0.84
8.35	36.9	0.24	176.14	338.1	1.1
12.05	40.0	0.26	219.6	421.6	1.39
16.35	42.8	0.27	263.3	505.5	1.9
20	43.8	0.28	332.9	639.1	2.07
25	45.4	0.29	484.5	930.2	2.5

**Table 2:** Irreversible volume shrinkage (Δ*V/V*_0_)_pl_, bulk density *ρ*, elastic moduli *K* and *E* and yield strength *σ*_el_ versus applied pressure *P* for AER50.

*P* (Mpa)	(Δ*V/V*_0_)_pl_ (%)	*ρ* (g·cm^−3^)	*K* (Mpa)	*E* (Mpa)	*σ*_el_ (Mpa)
0.1	0	0.245	51.04	97.9	0.87
1.75	0	0.245	50	96	0.82
2.76	0	0.245	49.1	94.3	0.73
5.77	1.63	0.249	45.9	88.1	0.79
8.85	4.9	0.257	43.4	83.3	0.7
14.66	12.6	0.28	44.3	85.1	0.74
20.1	26.4	0.333	57.02	109.5	0.91
24.7	37.7	0.393	92.2	177.1	1
30.3	44.7	0.442	122.7	235.6	1.65
34.8	47.5	0.466	143.5	275.5	1.75
42.4	54.8	0.542	217.5	417.6	2.64
48.4	56.5	0.563	231.7	444.8	2.74
52.5	60.2	0.616	295.8	567.9	3.63
61.7	63	0.663	330.3	634.1	4.14
70.5	66.74	0.737	398.8	765.6	4.57
80.7	68.14	0.769	443	850.5	5.7

**Table 3:** Evolution of sound velocity and elastic moduli *K* and *E* versus the bulk density for sintered gels (AER60).

*ρ* (g·cm^−3^)	*V* (m/s)	*K* (Mpa)	*E* (Mpa)
0.33	562	50.30	96.57
0.35	590	58,44	112.21
0.37	652	72.96	140.08
0.40	901	155.80	299.14
0.49	989	229.90	441.41
0.55	1202	379.90	729.41
0.65	1510	710.91	1364.95
0.75	1695	1033.59	1984.49

**Table 4:** Evolution of sound velocity and elastic moduli *K* and *E* versus the bulk density for compressed gels (AER60).

*ρ* (g·cm ^−3^)	*V* (m/s)	*K* (Mpa)	*E* (Mpa)
0.33	562	50.30	96.57
0.35	520	45.40	87.16
0.36	491	41.46	79.61
0.38	442	35.29	67.75
0.37	440	34.36	65.97
0.42	460	42.63	81.85
0.51	471	54.04	103.76
0.55	649	111.46	214.01
0.57	903	219.52	421.48
0.64	948	277.06	531.96
